# The effects of shoulder extension angle on elbow flexor hypertrophy in the cable curl exercise

**DOI:** 10.3389/fphys.2026.1750722

**Published:** 2026-03-31

**Authors:** Stian Larsen, Benjamin Sandvik Kristiansen, Nordis Østerås Sandberg, Andrea Bao Fredriksen, Roland van den Tillaar, Milo Wolf, Paul Alan Swinton, Hallvard Nygaard Falch

**Affiliations:** 1 Nord University Levanger, Levanger, Norway; 2 Lehman College, New York, NY, United States; 3 Robert Gordon University, Aberdeen, United Kingdom

**Keywords:** biceps brachii, muscle length, muscle thickness, resistance training, ultrasound

## Abstract

This within-participant randomized study compared elbow flexor hypertrophy following cable curls performed in a neutral *versus* maximally extended shoulder position, with resistance profiles and elbow extension range of motion (ROM) matched between conditions. Thirty untrained men participated, with one arm assigned to train at the individualized peak shoulder extension angle and the contralateral arm at a neutral position. Twenty-four participants completed the intervention, which consisted of six to eight weekly sets of unilateral cable curls to momentary failure over 10 weeks under supervised conditions. Elbow flexor muscle thickness was assessed with ultrasonography at 50% and 70% of humerus length before and after the intervention. A Bayesian framework was used to estimate the average treatment effect (ATE) and quantify the strength of evidence with Bayes factors (BF). Both conditions produced increases in muscle thickness of approximately 7%–9%. Posterior distributions of the ATE were centered close to zero (Proximal/ATE_Neutral:Peak_ = −0.40 [95% CrI: −1.06 to 0.26 mm]; Distal/ATE_Neutral:Peak_ = 0.21 [95% CrI: −0.25–0.65 mm]), with BFs indicating anecdotal to moderate evidence in favor of the null hypothesis. These findings suggest that when resistance profiles, elbow ROM, and effort are matched, altering shoulder extension angle in isolation does not appear to produce meaningful differences in elbow flexor hypertrophy in untrained men over 10 weeks.

## Introduction

Resistance training (RT), typically performed over several weeks to months, has consistently been shown to elicit small-to-moderate increases in skeletal muscle mass (Steele et al., 2023). Recently, Varovic et al. (2025) conducted a meta-analysis examining how mean muscle length during RT influences regional muscle hypertrophy. Pooling data from 13 studies, the authors observed a trend suggesting that resistance training at longer muscle lengths may promote greater distal compared to proximal hypertrophy. However, the authors emphasized that the precision of these estimates was limited due to the small number of studies and relatively low statistical power. Therefore, additional well-controlled studies are needed to refine effect estimates and strengthen the conclusions of future meta-analyses.

Moreover, it has been proposed that the hypertrophic response to training at longer muscle lengths may be muscle-specific (Ottinger et al., 2023). The elbow flexors, for example, have received increasing research attention over the past decade. Pinto et al. (2012) compared full range of motion (ROM) bicep curls with partial curls performed in a shortened position and found that both conditions increased elbow flexor thickness, with no significant differences between groups. As the biceps brachii is a biarticular muscle, its length is influenced by shoulder extension, suggesting that shoulder flexion/extension angle may affect the training stimulus and outcomes. Accordingly, several studies have compared incline curls to preacher curls (Kassiano et al., 2025; Zabaleta-Korta et al., 2023). For instance, Zabaleta-Korta et al. (2023) found that hypertrophy occurred only in the distal region of the elbow flexors following preacher curl training. Similarly, Kassiano et al., (2025) reported that preacher curls preferentially promoted distal elbow flexor hypertrophy, whereas incline curls favored elbow flexor proximal hypertrophy.

When elbow flexor thickness is measured at different sites along the humerus, distal measurements typically capture both biceps brachii and brachialis thickness, whereas proximal measurements primarily reflect the biceps brachii. This distinction arises because the brachialis originates from the distal half of the anterior humerus and as a monoarticular muscle, is unaffected by shoulder joint position. Consequently, findings from Kassiano et al. (2025) suggest that preacher curls, which impose greater torque at the beginning of the ROM, may promote distal hypertrophy *via* brachialis growth, whereas incline curls, which lengthen the biceps brachii through shoulder extension, may favor proximal biceps brachii hypertrophy. However, as muscle thickness was assessed collectively for the elbow flexors, it remains unclear whether these regional adaptations reflect biceps brachii or brachialis growth. Notably, Kobayashi et al. (2024) compared incline and preacher curls in a conference abstract, assessing biceps brachii and brachialis volumes separately using magnetic resonance imaging. They found that preacher curl training resulted in greater increases in brachialis volume, whereas incline curl training produced greater increases in biceps brachii volume. However, when comparing incline and preacher curls, both the resistance profile and muscle length differ. As the resistance profile differ between conditions, it’s challenging to distinguish how the shoulder flexion/extension angle itself impact muscle hypertrophy. Most of the previous studies comparing incline and preacher curl variations have manipulated shoulder angle together with changes in resistance profile and, in some cases, ROM. Consequently, it remains unclear whether reported regional differences in hypertrophy are driven by muscle length, by differences in torque distribution across the range of motion, or by an interaction between these factors.

Therefore, the aim of this study was to compare elbow flexor hypertrophy between a maximized individualized shoulder extension angle and a neutral shoulder position, while matching resistance profiles across conditions. We hypothesized that, because the brachialis is unaffected by shoulder joint angle, similar torque demands would result in comparable brachialis hypertrophy, whereas the biceps brachii would undergo greater lengthening with increased shoulder extension, potentially leading to greater overall elbow flexor hypertrophy.

## Methods

### Risk of bias and transparent considerations

The study was preregistered prior to study start on the Open Science Framework (https://osf.io/azfrc). To reduce potential biases, this study aimed to follow the Standards Method for Assessment of resistance training in Longitudinal Design (SMART-LD) check-list proposed by Schoenfeld et al. (2023), which resulted in a final grading of 19 out of 20 points (good quality). Also, the research team standardized training frequency, set volume, repetition volume, rest intervals between limbs, proximity-to-failure, elbow flexion ROM, lifting durations in both the eccentric and concentric phase as these training variables could influence the RT stimuli and potentially cofound the results (Coratella, 2022). Additionally, all participants trained both leg extensions and standing calf raises using the Smith machine, which were part of other studies that have already been published elsewhere (Larsen et al., 2024; Larsen et al., 2025). Despite preregistration of the study methods prior to the start of the intervention, some methodological adjustments were made following the final pilot test: (a) Although the intervention aimed to maintain a constant shoulder extension angle for the peak° limb throughout the training period, this was not fully achievable as participants self-regulated their movement patterns during sets, together with some daily variations in shoulder extension flexibility. (b) Ultrasonography was originally planned to be performed weekly, but this was not feasible due to sonographer availability constraints. (c) The initial plan was to measure separate muscle thickness for the biceps brachii and brachialis; however, this was changed after the pilot testing to combined elbow flexor muscle thickness, as this approach yielded less measurement error.

### Participants

**FIGURE 1:**
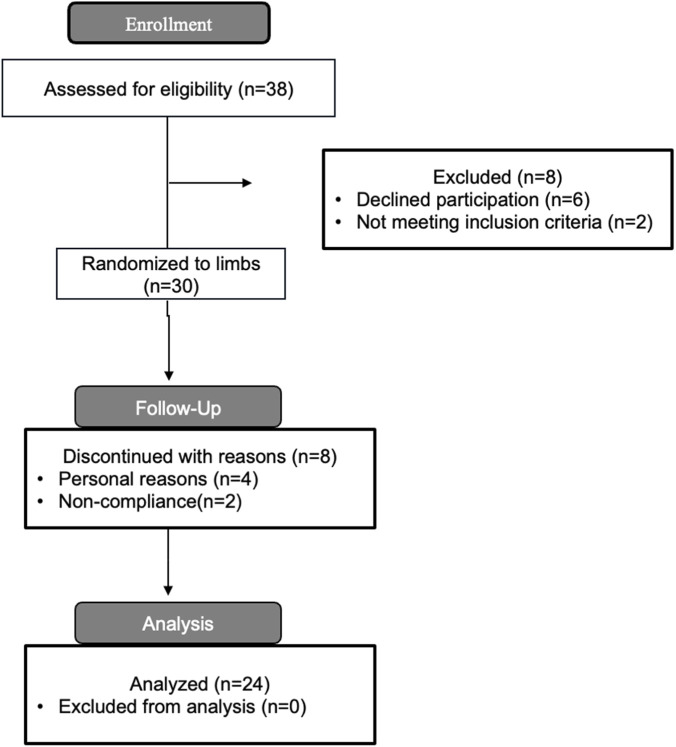
Flow diagram of participant enrollment, allocation, follow up, and analysis.

Although conducting *a priori* power analyses is a common practice to determine the sample size needed to detect the smallest meaningful effect with adequate statistical power, practical limitations such as available resources often dictate the final number of participants (Lakens, 2022). Therefore, we aimed to recruit as many participants as feasibly possible within our resource constraints to maximize statistical power. Additionally, employing a within-participant design increases statistical power by reducing between-subject variability, as each participant serves as their own control, thereby improving the precision of effect estimates and enhancing the ability to detect true differences between conditions (Robinson et al., 2024). Ultimately, we succeeded in enrolling a sample of 30 healthy, untrained, but physically active men aged between 18 and 50 years (See Figure 1). Inclusion criteria were as follows: 1) no self-reported prior use of anabolic steroids, 2) no previous resistance training experience defined as fewer than one resistance training session per week during the 6 months preceding study start, and 3) absence of cardiorespiratory or musculoskeletal disorders that could affect study outcomes. Furthermore, participants were required to attend more than 85% of the scheduled resistance training sessions to be included in the statistical analyses. Consequently, 24 participants were included in the final analyses (mean age: 31.9 ± 5.6 years; body mass: 84.4 ± 11.6 kg; height: 180.1 ± 5.8 cm; see Figure 1). Because the statistical power of this study may be limited, efforts have been made to ensure that the data collected from this study may be easy to use in future meta-analytical frameworks.

All participants received dietary guidance recommending a daily protein intake exceeding 1.6 g per kg of body mass, based on Morton et al. (2018). Participants were also instructed to maintain their habitual dietary patterns throughout the intervention. The study procedures were thoroughly explained to each participant both verbally and in writing. Informed consent was obtained from all participants prior to inclusion. The study protocol was submitted to the Regional Committees for Medical and Health Research Ethics and was deemed exempt from full review (application number 696,927). Additionally, the research project received approval from SIKT–The Norwegian Agency for Shared Services in Education and Research (reference number: 125,855) and was conducted in accordance with the latest version of the Declaration of Helsinki.

### Procedures

**FIGURE 2:**
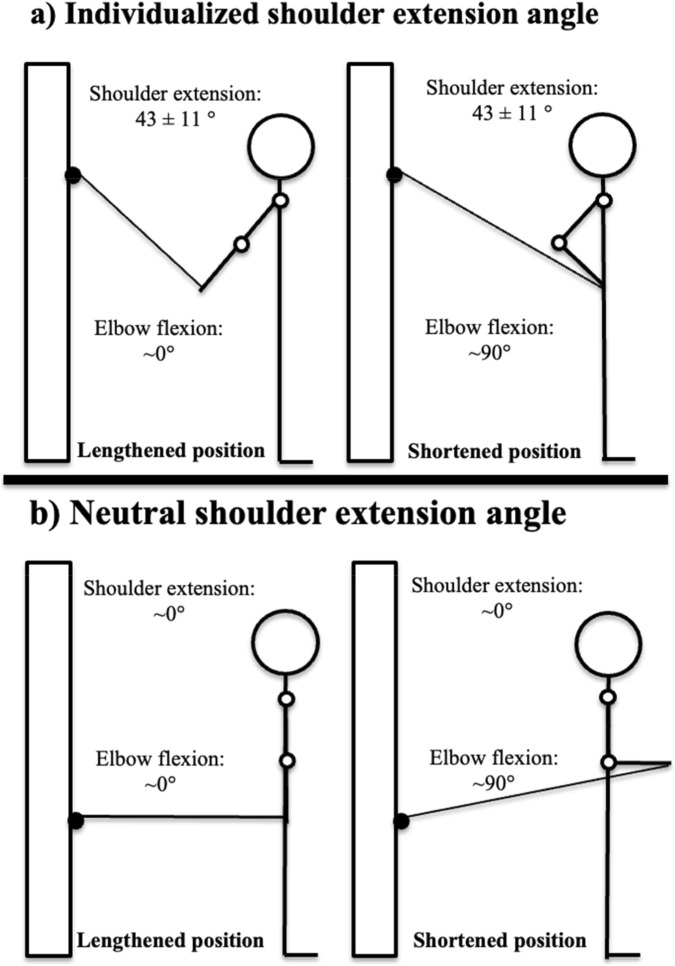
Illustration of the mean (SD) shoulder extension angle and corresponding elbow flexion range of motion for the two experimental conditions assessed at baseline. **(a)** Individualized peak shoulder extension condition, showing the mean (SD) shoulder extension angle in both the lengthened (elbow near 0^°^) and shortened (elbow near 90^°^) positions. **(b)** Neutral shoulder extension condition, showing a shoulder position close to 0° in both the lengthened (elbow near 0^°^) and shortened (elbow near 90^°^) positions.

This study employed a within-participant design, with the right and left upper limbs randomized *via* www.randomizer.org to one of two shoulder extension angles: an individualized peak shoulder extension angle (peak°) or a neutral shoulder extension angle (neutral°; see Figure 2). Following two baseline ultrasound assessments, participants completed a familiarization session, and subsequently trained biceps curls unilaterally for 10 weeks. All training sessions were supervised by an experienced research team, and muscle thickness of the elbow flexors was measured before and after the intervention using ultrasound imaging. The data collection was conducted between January 2024 and March 2024 in Levanger.

To increase statistical power (Swinton, 2024) and quantify measurement error for the ultrasound measurements, participants met twice for baseline- and postintervention testing. Instructions for all participants were made to not engage in training or vigorous physical activity 96 h before the baseline- and postintervention tests. Also, participants were instructed to avoid food 2 hours and caffeine 8 hours before ultrasound measurements and we did our best to book the ultrasound tests at similar times at the day. Ultrasound measurements of the elbow flexors (biceps brachii plus brachialis) were obtained using Echo Wave software (Telemed, Latvia) with a 60-mm probe and a scanning frequency of 9 MHz. The same sonographers performed all assessments at both baseline and post-test. Upon arrival, participants were placed in a supine position and remained still for 10 min prior to measurement. Reference lines were marked on the skin using a marker, and images were taken of these lines for all participants to increase the likelihood for accurate comparisons between baseline- and postintervention testing. All reference images and muscle thickness measurements were stored on a password-protected external flash drive. A thin layer of Chemolan transmission gel (Chemodis, DA, Alkmaar, Netherlands) was applied to the skin over the targeted muscle area. In addition to the elbow flexors, muscle thickness measurements were taken for the rectus femoris, vastus lateralis, and medial gastrocnemius for use in two separate studies. For the elbow flexors, measurements were taken at 50% and 70% of the distance from the lateral epicondyle of the humerus to the acromion, in line with the procedures by Sato et al. (2021) where elbow flexor thickness were measured from the humerus to the inner edge of the fascia. The ultrasound probe was positioned perpendicular to the muscle tissue interface. Two researchers conducted all MT assessments at both baseline and post-test; one operated the probe while the other captured the image. When image quality was deemed satisfactory, three images were taken at both the 50% and 70% sites. The average of these three measurements were used as a proxy for muscle size. To assess measurement error and reliability, typical error and intraclass correlations were calculated for all participants between baseline tests one and two and postintervention tests one and two. Typical error for all sites was less than 0.54 mm (range: 0.37–0.54 mm), and the intraclass correlations were all above 0.96 (range: 0.96–0.99).

The resistance training intervention was performed over a 10-week period, with a minimum of 48 h between each session. The biceps curl exercise was performed unilaterally using a Gymleco 225 cable crossover multi-gym unit (Gymleco, Eskilstuna, Sweden). Participants completed two sessions per week, performing three sets per limb in each session. From week 5, session 2, the number of weekly sets increased from six to eight total sets per limb. Each set consisted of 10–20 repetitions to momentary concentric failure, defined as the inability to complete the concentric phase of a repetition despite attempting to do so (Steele et al., 2017). A double progression method was employed, wherein participants increased the load by 0.25 kg when they achieved more than 20 repetitions in the first set of an exercise, ensuring that effort remained within the designated repetition range. Training intensity was standardized by having all sets terminated at momentary failure, with approximately 2 min of rest between sets and 30 s between limbs. The limb order varied across sessions to avoid systematic bias due to training sequence. Each participant trained both arms under two different conditions: neutral° shoulder extension and peak° shoulder extension. During both conditions, the supervisors provided manual elbow support by placing their hands just behind the participants elbow to ensure stability. The right and left upper limbs were randomized using www.randomizer.org. In the neutral° condition, the shoulder was aligned parallel to the torso. In the peak° condition, the shoulder was positioned at approximately 5°–10° less than the participant’s individual maximal shoulder extension angle, which was determined during a familiarization session using an electronic goniometer (EasyAngle, Stockholm, Sweden). Participants stood upright in the anatomical position and performed three active shoulder extensions of the glenohumeral joint. The average value from these trials was used to define the individualized peak° position. Participants gradually worked up to their 10–20 repetition maximum load for each arm and condition during the familiarization session, and this load was used as the starting point for the intervention. Participants were instructed to maintain shoulder movement strictly within the sagittal plane, avoiding abduction in the frontal plane. Although the original aim was to limit scapular elevation and rotation, this was permitted throughout the intervention to ensure consistent execution, as most participants struggled to perform shoulder extension without scapular involvement during familiarization. Elbow flexion was standardized between 0° and 90° and kept consistent between conditions. The cable height and body positioning were individually adjusted to create a similar descending resistance profile across both experimental conditions as seen in Figure 2. Volume load, defined as sets × repetitions × load, was documented for each participant and condition. Repetition tempo was standardized across all sessions where participants were instructed to perform the concentric phase as fast as possible, and the eccentric phase over approximately 2 s. A controlled stop was required at both the top and bottom positions of the movement. Verbal encouragement and feedback were provided throughout all sessions to ensure maximal effort and adherence to the technical standards. All training sessions were conducted at the same facility using the same equipment and were supervised by experienced personal trainers (each with at least a bachelor’s degree in sports science and Norwegian personal trainer certification). All supervisors received detailed instruction from the lead researcher and participated in two pilot sessions to standardize technique and protocol adherence. Supervision was conducted at a 1:1 to 2:3 trainer-to-participant ratio. Every session was documented, including limb condition, load, number of repetitions, and cable settings. Participants were allowed to perform additional resistance training according to a standardized program provided by the research team, including exercises such as bench press, shoulder press, cable pullover, triceps extensions, leg curl, and abdominal exercises. Importantly, none of these exercises directly trained the elbow flexors.

## Statistics

Analyses were conducted in R (version 4.4.0) using a Bayesian framework, which allowed incorporation of informative priors from previous studies. This approach enabled inferences to be drawn in terms of probability and allowed assessment of the strength of evidence for the null *versus* alternative hypothesis (Schad et al., 2022). Both univariate and multivariate linear mixed effects models were employed, with random effects accounting for the within participant design and the repeated measures (Magezi, 2015). The effects of shoulder extension angle (peak° *versus* neutral°) upon muscle thickness increases from baseline-to post-intervention testing was quantified through Average Treatment Effects (ATE). Within-condition effects were also quantified to evaluate the effectiveness of each training condition separately. These effects were compared against thresholds specific to strength and conditioning research (Swinton et al., 2022). Inferences were based on: (a) posterior distributions of the ATE with corresponding credible intervals, (b) Bayes factors (BF) quantifying the strength of evidence for a non-zero ATE (alternative hypothesis) *versus* zero ATE (null hypothesis), and (c) posterior probabilities that the ATE was practially meaningful, defined relative to a region of practical equivalence (ROPE). The ROPE was defined as a difference of −3 to +3%, consistent with previous research (Varovic et al., 2025). The ROPE was converted to the absolute (mm) scale using the pooled mean, and for both measurements sites the resulting ROPE bounds exceeded the typical error of measurement. Qualitative labels that expressed strength of the evidence for the two different hypotheses were adopted (Lee and Wagenmakers, 2014). A structured Bayesian workflow was followed, consisting of: (a) incorporating informative priors from relevant meta-analyses (Swinton et al., 2022), (b) performing simulation-based calibration of BFs (Schad et al., 2022), (c) evaluating priors with prior predictive checks, (d) assessing stability of estimates *via* repeated iterations with the same data, and (e) evaluating posterior distributions with posterior predictive checks and sensitivity analyses (including non-informative priors). The primary informative priors were placed on the model intercept, representing the expected improvement in the neutral group, and on the ATE. Both priors were specified as normal distributions centered at zero, with standard deviations of 0.44 and 0.4 × the baseline measurement standard deviation for the interept and ATE, respectively. All models were estimated using the brms package interfaced with Stan (Bürkner, 2017). Bayes factors were estimated *via* the bridge sampling algorithm (Gronau et al., 2017). Acceptable analytical precision was obtained, with credible intervals approximately spanning the width of each ROPE. Finally, to improve the transparency, accuracy, and potentially, replication in our analyses, the WAMBS-checklist (When to worry and how to Avoid Misuse of Bayesian Statistics) including full information on priors was used based on the work of Depaoli and Van de Schoot (2017), and reported in Supplementary File 2.

## Results

### Attendance

The average attendance was 19.2 of 20 RT sessions. Twenty-four out of the thirty participants that started the intervention completed the RT intervention. Two participants dropped out due to non-compliance, whereas four participants dropped out due to personal reasons. Because this was a within-participant design, attendance was necessarily equal across conditions.

### Muscle morphology

Univariate analyses indicated evidence in favor of the null hypothesis for the proximal region (BF = 0.56; anecdotal evidence for the null) and distal region (BF = 0.24; moderate evidence for the null). Posterior distributions of the ATE were centered close to zero and lay almost entirely within the ROPE (Proximal/ATE_Neutral:Peak_ = −0.40 [95% CrI: −1.06 to 0.26 mm], P (ATE within ROPE) = 0.962; Distal/ATE_Neutral:Peak_ = 0.21 [95% CrI: −0.25–0.65 mm], P (ATE within ROPE) > 0.999). When combining data within a multivariate framework, evidence also favored the null hypothesis (BF = 0.17; moderate evidence for the null). Within-group changes are presented in Table 1, with point estimates indicating that both interventions mostly produced effects of a medium magnitude (∼7.5–13.5% improvement, Swinton et al., 2022) across both regions.

**TABLE 1:** Descriptive summary of pre- and post-intervention values (mean ± SD).

	Peak° (n = 24)			Neutral° (n = 24)		
Variable	Pre	Post	Δ%	Pre	Post	Δ%
Elbow flexor distal (mm)	34.6 ± 4.1	37.1 ± 4.3	7.2 ± 3.1	35.0 ± 3.7	37.2 ± 4.0	6.3 ± 2.8
Elbow flexor proximal (mm)	27.2 ± 4.3	29.2 ± 4.4	7.9 ± 6.2	27.2 ± 4.0	29.7 ± 4.6	9.2 ± 6.7

#### Volume load

**FIGURE 3:**
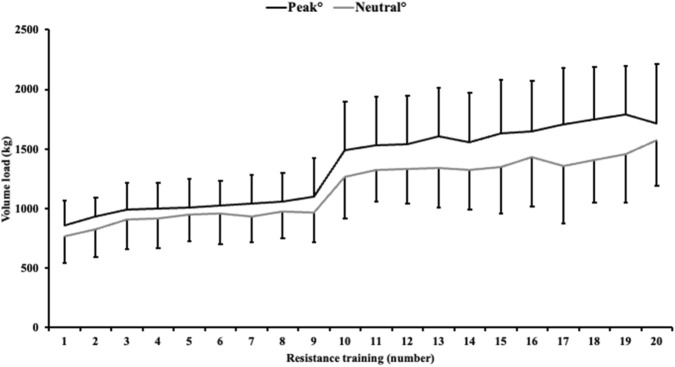
Volume load per resistance training session shown as mean +SD for the peak° condition and mean–SD for the neutral° condition.

The volume load increased from 856 ± 213 kg and 771 ± 230 kg in the first RT session to 1102 ± 320 kg and 969 ± 254 kg in the ninth RT session for the peak° and neutral° conditions, respectively (Figure 3). When increasing set volume from three to four sets, volume load increased from 1490 ± 405 kg and 1268 ± 355 kg in RT session ten to 1715 ± 496 kg and 1573 ± 379 kg in the last RT session, for the peak° and neutral° conditions, respectively.

## Discussion

This study investigated the effect of shoulder extension angle on elbow flexor hypertrophy using a within-participant design with matched resistance profiles. Both conditions produced medium-magnitude increases in muscle thickness (peak° distal and proximal Δ = 7.2 and 7.9%; neutral° distal and proximal Δ = 6.3 and 9.2%), but no meaningful differences were observed between neutral and individualized peak shoulder extension training, with analyses providing anecdotal to moderate evidence in favor of the null hypothesis. Consistent with this interpretation, the posterior distributions of the average treatment effects were centered close to zero and lay almost entirely within the predefined region of practical equivalence, indicating a high probability that any true difference between conditions was too small to be considered practically meaningful. A multivariate analysis likewise provided evidence in favor of the null hypothesis, supporting the interpretation that both conditions produced improvement of similar magnitudes.

The present findings align with those of Attarieh et al. (2025) who recently compared two unilateral cable curl variations in untrained men: Bayesian curls (shoulder extended ∼50°) and preacher curls (shoulder flexed ∼50°). Using a within-subject design with matched resistance profiles over 9 weeks, they observed no significant differences in biceps brachii thickness across all measured regions (proximal, mid, distal), as well as in brachialis thickness. Bayesian curls resulted in ∼9% increases in biceps brachii thickness, while preacher curls produced ∼7% gains, with no statistically significant differences between conditions outcomes. similar to those of the present study.

Despite altering biceps brachii muscle length *via* shoulder extension and that the participants consistently lifted greater loads when training bicep curls with the peak° condition, this did not result in greater elbow flexor muscle thickness increases. This was surprisingly as previous studies have suggested that increasing shoulder extension may increase the force production capability of the bicep brachii (Attarieh et al., 2025; Murray et al., 2000), which logically occurred in this study since the participants consistently lifted greater loads with the peak° condition during the RT intervention even if the external moment arm and lifting tempo was standardized between conditions. Interestingly, the participants in the present study were weaker in the neutral° (shortened) condition which is opposite to what Attarieh et al. (2025) observed, even if they in their study shortened the biceps brachii to a greater extent in the preacher curl condition compared to what the participants in the current study did. It is speculated that the extra support from the preacher curl bench in their study may have increased stability and increased the capability to increase force as the posterior part of the humerus had support from the preacher curl which again adds an extra reaction force and more stability which may potentially have impacted the force production in the preacher curl condition. However, in the present study no differences in support were provided between conditions. Thus, our study adds to Attarieh et al. (2025) where we demonstrated that training biceps curl with a greater shoulder extension together with extra volume load did not augment elbow flexor hypertrophy.

Moreover, as elaborated in the introduction, other laboratories have compared different elbow flexor exercises similar to those used in the present study (Kassiano et al., 2025; Kobayashi et al., 2024; Zabaleta-Korta et al., 2023) and observed regional differences in elbow flexor hypertrophy between conditions. Nevertheless, when resistance profiles are standardized and only biceps brachii muscle length is manipulated *via* shoulder extension or flexion angle, the present longitudinal evidence does not support that altering shoulder position affects distinct biceps brachii or brachialis growth (Attarieh et al., 2025) or combined elbow flexor growth. However, there is considerable uncertainty regarding the true population parameters, as the credible intervals cross zero. Therefore, the findings should be considered preliminary, and future laboratories should attempt to replicate these results with open datasets to enable meta-analytical interpretations.

Anecdotally, we observed that some of the participants repeatedly failed at higher concentric and angular elbow extension velocities in both conditions. It is speculated that this occurred due to the high external moment arm coupled with a small internal bicep brachii moment arm. For instance, the biceps brachii moment arm is suggested to decrease with increased elbow extension (Murray et al., 1995). Consequently, we postulate that setting up a resistance profile that peaks in full elbow extension could decrease muscle growth since the participants were not able to pursue some of the sets to slower angular velocities that may be associated with a decreased proximity-to-failure. However, this speculation needs further investigation to draw any firm conclusions.

### Limitations

Several limitations should be noted. First, the sample consisted of untrained men, a population known to respond robustly to a wide variety of resistance training stimuli. This responsiveness may mask subtle differences between training modalities. Thus, the difference between neutral° and peak° positions may not have been large enough to produce divergent outcomes within the constraints of a short intervention and modest sample size. Also, the present findings are limited to untrained men and may not generalize to women or resistance-trained individuals, whose hypertrophic responses and sensitivity to manipulations of muscle length may differ. Second, while the matched resistance profile is a methodological strength, it may also have caused premature set terminations in both conditions since the external moment arms peaked in the most lengthened position. Future studies should therefore consider applying a more conservative resistance profile. Third, although shoulder joint angle was standardized, participants inevitably varied in their ability to maintain this position across repetitions. Fourth, the study measured whole elbow flexor thickness rather than differentiating between biceps brachii and brachialis, which limits our ability to draw muscle-specific conclusions. Therefore, the present findings apply to elbow flexors as a group rather than to individual muscles. Future research should therefore employ imaging modalities capable of distinguishing these muscles. Finally, the relatively short duration of the intervention, the moderate sample size, and the specificity of the training model, consisting of single-joint elbow flexion with a descending resistance profile, should be considered when interpreting the generalizability of the findings.

### Practical applications

The present findings suggest that training elbow flexors with either a neutral or maximally extended shoulder position results in comparable hypertrophic outcomes when key training variables (intensity of effort, volume, resistance profile) are controlled. For most recreational lifters or novice clients, either configuration may be effective options, and exercise selection should therefore be based on personal preferences.

## Conclusion

This study observed no differences in proximal or distal elbow flexor hypertrophy between neutral and maximally extended shoulder positions when resistance profiles were matched and training was performed to momentary failure. Thus, both modalities are effective for increasing overall elbow flexor muscle thickness.

## Data Availability

The datasets presented in this study can be found in online repositories. The names of the repository/repositories and accession number(s) can be found in the article/Supplementary Material.
