# Supplementary material for: The effects of shoulder extension angle on elbow flexor hypertrophy in the cable curl exercise
Source: Front Physiol. 2026 Mar 31;17:1750722. doi: 10.3389/fphys.2026.1750722 (PMC13076143; doi:10.3389/fphys.2026.1750722)
Supplement: Supplementary file 2 [file DataSheet1.docx]

**Supplementary file 3.**

The following supplementary file provides presents the WAMBS (When to worry, and how to Avoid the Misuse of Bayesian Statistics) checklist as a diagnostic tool that was used to assess prior distributions, the estimation process, and the influence of priors for analysis of body composition outcomes; and overview of simulation based calibration performed on Bayes factors. The following provides details of the WAMBS checklist and how it was used.

| **The WAMBS-Checklist****When to worry, and how to Avoid the Misuse of Bayesian Statistics** **Depaoli & Van de Schoot (2017)(1)** | |
| --- | --- |
| **To be checked before estimating** |  |
| **Point 1:** Do you understand the priors? | Analyses were conducted using linear mixed effects models with random effects included to account for the within participant design such that participant intercepts were included, and also modelled variation in change across conditions. Informative priors were included to model within-condition change based on likely improvements in strength and conditioning (2), and average treatment effects (3). Default weakly informative priors were used for all variance parameters including random effects. |
| **To be checked after estimation** |  |
| **Point 2:** Does the trace-plot exhibit convergence? | Trace-plots were examined and all Rhat values were reported. |
| **Point 3:** Does convergence remain after doubling the number of iterations? | Trace-plots were examined and bias for group parameter presented as a percentage 100*(original – doubling)/original reported. |
| **Point 4:** Does the histogram have enough information? | Plot of histogram for all parameters presented. |
| **Point 5:** Do chains exhibit autocorrelation? | Plot of autocorrelation for all parameters presented. |
| **Point 6:** Do posterior distributions make sense? | In all cases yes |
| **Understanding influence of priors** |  |
| **Point 7:** Do different variance priors influence the results? | Sigma was modelled using weakly-informative Half-t distributions with 3df. As a check, informative gamma priors with shape k based on outcome and scale $\theta$ set to 1. Bias in group parameter was presented. |
| **Point 8:** Is there a notable effect of the prior when compared with non-informative priors? | As a check, models were conducted with all default weakly informative priors and bias in group parameter presented. |
| **Point 9:** Are the results stable from a sensitivity analysis? | Checked in each case, use of informative priors tended to reduce point estimate and tails of ATE. |
| **After interpretation of results** |  |
| **Point 10**: Is the Bayesian way of interpreting and reporting model results used? *(a) Also report on: missing data, model fit and comparison, non-response, generalizability, ability to replicate, etc.* | Reporting of results combining in-text and supplementary file was done in a comprehensive and systematic manner, focussing on the ATE. Posterior probabilities were also used to summarise likely differences and Bayes factors used to quantify strength of evidence for the two hypotheses. |

(1) Depaoli S, van de Schoot R. Improving transparency and replication in Bayesian statistics: The WAMBS-Checklist. Psychol Methods 2017 June 01;22(2):240-261.

(2) Swinton PA, Burgess K, Hall A, Greig L, Psyllas J, Aspe R, et al. Interpreting magnitude of change in strength and conditioning: Effect size selection, threshold values and Bayesian updating. J Sports Sci 2022 September 01;40(18):2047-2054.

(3) Swinton PA, Murphy A. Comparative effect size distributions in strength and conditioning and implications for future research: A meta-analysis. SportRxiv 2022:DOI: 10.51224/SRXIV.202.

**WAMBS: Univariate elbow flexor thickness**

Sample data

**Distal Proximal**


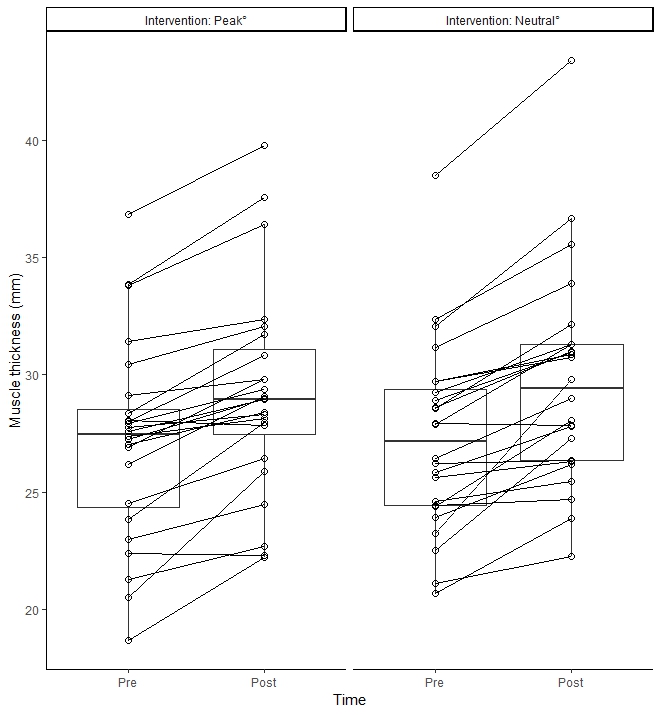


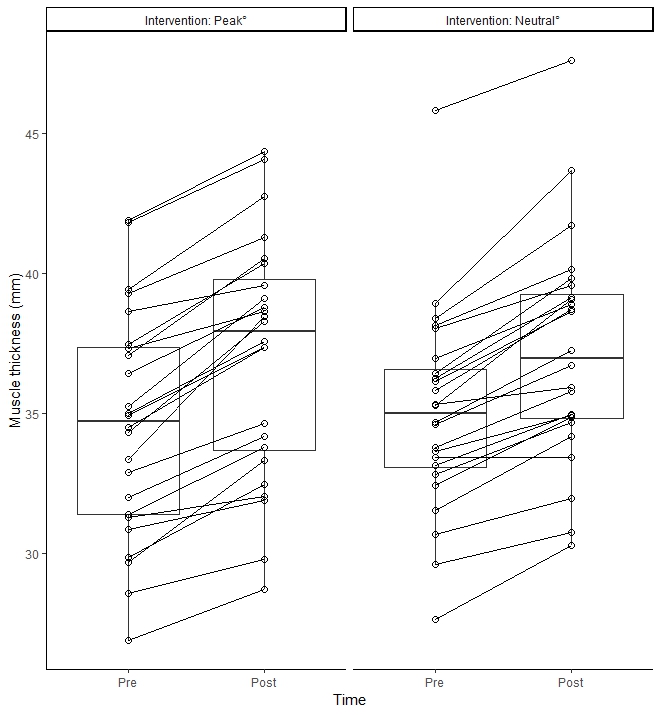


**WAMBS: Univariate elbow flexor thickness**
Understanding priors

| Outcome | Elbow flexor distal | Elbow flexor proximal |
| --- | --- | --- |
| Pre-Post Change | Normal(1.7,1.7^2^) | Normal(1.8,1.8^2^) |
| Average Treatment Effect | Normal(0,1.5^2^) | Normal(0,1.65^2^) |
| Prior predictive check | 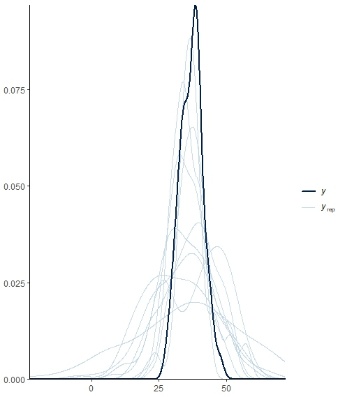 | 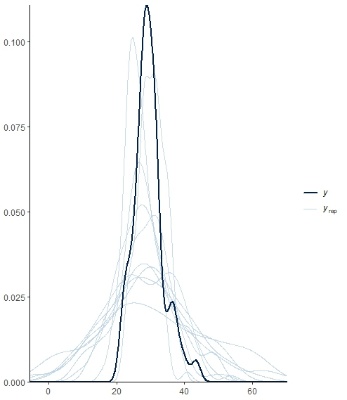 |

Estimation

| Rhat values | All equal 1.0 | All equal 1.0 |
| --- | --- | --- |
| Bayes factors iterations | 0.24; 0.24; 0.25; 0.24 | 3447; 3470; 3449; 3500 |
| Bias doubling iterations (ATE) | 0.1% | -0.2% |
| Posterior predictive check | 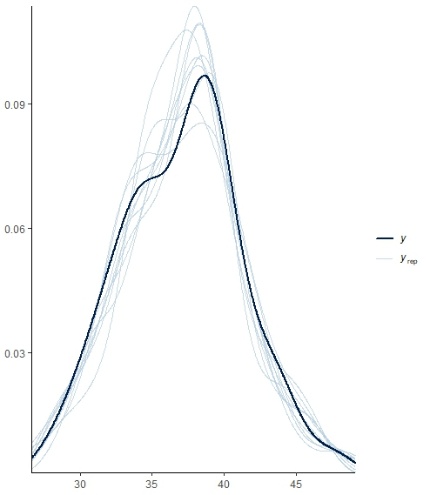 | 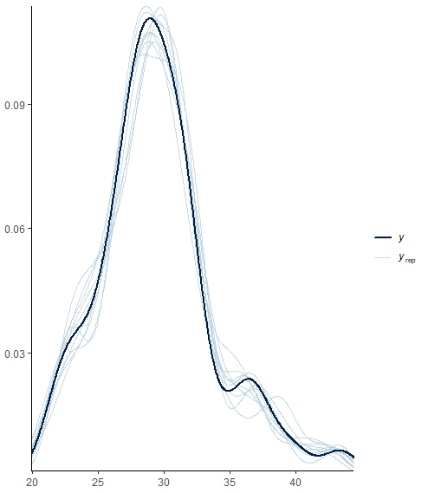 |
| Autocorrelation | 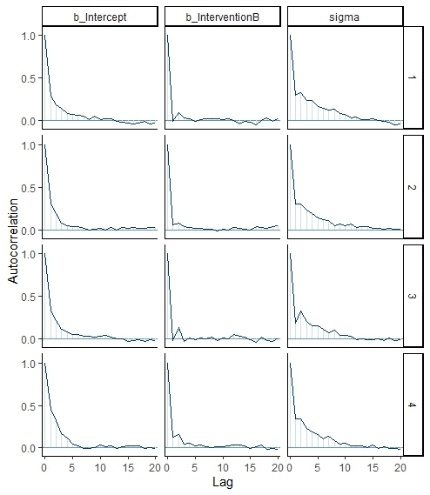 | 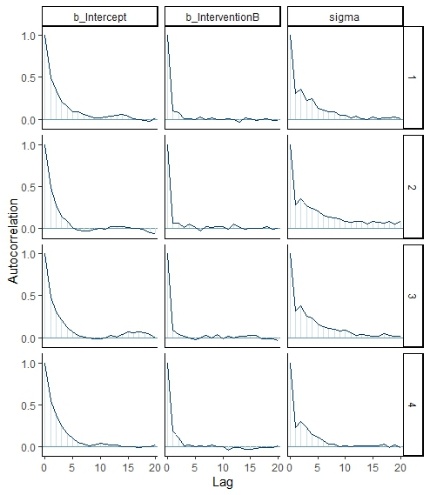 |

Influence of priors

| Bias different specification variance (ATE) | Gamma(2,1)  -0.5% | Gamma(2,1)  -0.7% |
| --- | --- | --- |
| Bias after non-informative priors (ATE) | -3.1% | 4.3% |

**Simulation based calibration of Bayes factors**

Simulation based calibration (SBC) of Bayes factors was performed to assess whether Bayes factors obtained for this study were likely to be appropriate. Artificial data were simulated (m=500 samples) based on the number of participants and design of the study, with parameters selected and distributed to match our a priori expectations.

Intercept = N(10,1^2^)

Pre to post = N(1.25, 0.2^2^)

Baseline difference = N(0, 0.1^2^)

ATE = N(0, 0.75^2^).

For half of the simulations the ATE was set to 0, and for the other half set to the random draw from N(0,0.75^2^). For each iteration, the model with the research hypothesis (ATE$\neq$0, H_1_) and null hypothesis (ATE=0, H_0_) were fit and the Bayes factor estimated.

The distribution of the Bayes factors given the true hypothesis were:


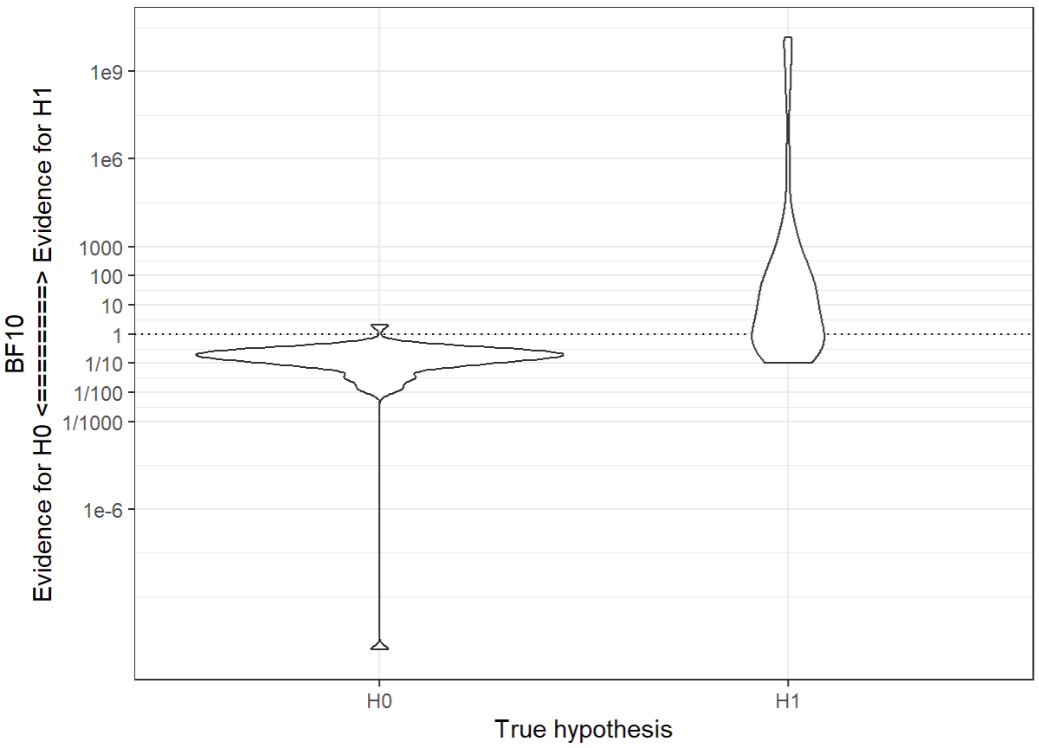


The SBC started with equal probability (0.5) applied to the two hypotheses. Following the 500 simulations, the posterior model probabilities were calculated and this was used to quantify how likely each model was chosen a posteriori on average. These were compared to the prior model probabilities that were used to simulate the data (48.2 [95%CI: 44.3 to 53.9]).

We also calculated the average posterior probabilities as a function of the true hypothesis:

| True hypothesis | pH_0_ | pH_1_ |
| --- | --- | --- |
| H_0_ | 85 | 15 |
| H_1_ | 28 | 72 |
